# Simultaneous 3D Visualization of the Microvascular and Neural Network in Mouse Spinal Cord Using Synchrotron Radiation Micro-Computed Tomography

**DOI:** 10.1007/s12264-021-00715-7

**Published:** 2021-06-19

**Authors:** Liyuan Jiang, Chengjun Li, Miao Li, Xianzhen Yin, Tianding Wu, Chunyue Duan, Yong Cao, Hongbin Lu, Jianzhong Hu

**Affiliations:** 1 Department of Spine Surgery, Xiangya Hospital, Central South University, Changsha, 410008 China; 2 National Clinical Research Center for Geriatric Disorders, Xiangya Hospital, Central South University, Changsha, 410008 China; 3 Key Laboratory of Organ Injury, Aging and Regenerative Medicine of Hunan Province, Changsha, 410008 China; 4 Department of Sports Medicine, Xiangya Hospital, Central South University, Changsha, 410008 China; 5 Xiangya Hospital-International Chinese Musculoskeletal Research Society Sports Medicine Research Centre, Changsha, 410008 China; 6 Hunan Engineering Research Center of Sport and Health, Changsha, 410008 China; 7 Center for Drug Delivery Systems, Shanghai Institute of Materia Medica, Chinese Academy of Sciences, Shanghai, 201203 China

**Keywords:** Srμct, 3D, High-resolution, Neurovascular, Spinal cord

## Abstract

Effective methods for visualizing neurovascular morphology are essential for understanding the normal spinal cord and the morphological alterations associated with diseases. However, ideal techniques for simultaneously imaging neurovascular structure in a broad region of a specimen are still lacking. In this study, we combined Golgi staining with angiography and synchrotron radiation micro-computed tomography (SRμCT) to visualize the 3D neurovascular network in the mouse spinal cord. Using our method, the 3D neurons, nerve fibers, and vasculature in a broad region could be visualized in the same image at cellular resolution without destructive sectioning. Besides, we found that the 3D morphology of neurons, nerve fiber tracts, and vasculature visualized by SRμCT were highly consistent with that visualized using the histological method. Moreover, the 3D neurovascular structure could be quantitatively evaluated by the combined methodology. The method shown here will be useful in fundamental neuroscience studies.

**Supplementary Information:**

The online version contains supplementary material available at 10.1007/s12264-021-00715-7.

## Introduction

Since the spinal cord is the main pathway for neural signals connecting the central nervous system (CNS) to the peripheral nervous system (PNS), diseases of the spinal cord can interrupt these connections, resulting in impairments of sensory and motor functions [1]. Neurons, nerve fibers, and blood vessels are the main structures that constitute the spinal cord microenvironment. Their structural changes are frequently associated with the occurrence and development of spinal cord disorders [2–4]. Therefore, exploring the morphology of the neurovascular microstructure is of great importance for understanding the pathogenesis and development of diseases and evaluating the effectiveness of treatments.

Both the neural network and vascular architecture in the spinal cord are very complicated. To acquire morphological information, scientists have developed various methods to explore the neurovascular morphology of the spinal cord. Current morphological studies of neurons and vasculature mostly rely on a two-dimensional (2D) histological method, which can only provide impressive 2D images of the neural system or vessels. This yields incomplete spatial coverage and requires destructive sample preparation, leading to potential misinterpretation of data. In recent decades, two-photon excitation microscopy has been developed as an advanced imaging tool that can provide crucial 3D information. Nevertheless, its use is limited by the penetration depth and the size of the region of interest [5]. Micro-optical sectioning tomography is another novel and powerful tool developed in recent years to provide 3D morphological information [6]. However, its sample preparation is destructive and time-consuming. In clinical settings, magnetic resonance imaging and X-ray computed tomography are widely used 3D techniques that have been used for a long time. However, their spatial resolution limits the possibility of further visualizing neuronal networks and capillaries.

Thus, a more efficient and high-resolution 3D nondestructive imaging technique is urgently needed to investigate detailed neurovascular structures. High-resolution synchrotron radiation micro-computed tomography (SRμCT) is particularly suitable for low-absorbing biomedical samples [7]. Fratini *et al*. first achieved simultaneous submicrometric 3D imaging of the microvascular network and the neuronal system in mouse spinal cord using synchrotron radiation X-ray phase-contrast tomography (SRXPCT) [8]. Then, many researchers successfully acquired 3D images of neurons and/or microvessels in the CNS using SRXPCT [7, 9–15]. Among these studies, Töpperwien *et al*. evaluated the influence of different embedding media on 3D imaging of neuronal cytoarchitecture in the brain by using a synchrotron-based holotomography setup and suggested that different embedding media have different advantages [12]. Most recently, Barbone *et al*. even demonstrated the use of X-ray phase-contrast micro-CT for high-resolution 3D visualization of embalmed human spines [16], highlighting that it has excellent advantages for 3D visualization of the neural system and vasculature. Although the 3D neuronal soma and a part of the capillary can be well visualized using SRXPCT, there are still many aspects that require further improvement to obtain more detailed and high-resolution (sub-micron) 3D morphological information. For example, it is challenging to distinguish axons and dendrites from the surrounding tissue and obtain the detailed morphology of a single neuron with its soma using SRXPCT, since the refractive index of axons and dendrites is very similar to that of the surrounding tissue [7, 9, 10]. It is also difficult to obtain 3D images of nerve fiber tracts in white matter using SRXPCT, and there are still few 3D morphological depictions of nerve fibers in previous studies. In addition, tissue shrinkage, vascular deformation, and vascular collapse during the process of sample preparation are also unsolved problems, which may lead to low-quality images and inaccurate interpretations [17, 18]. Overall, low-quality images largely restrict the quantitative analysis of 3D neuronal and vascular morphology. Therefore, further improvements are needed to obtain more detailed and accurate images and to quantify the 3D morphology of neural and vascular networks.

Recently, Barbone *et al*. suggested that choosing proper fixation protocols for optimal preservation of the structure of nervous tissue is of importance in achieving effective and targeted neuroimaging using SRμCT [19]. SRμCT combined with angiography has been proposed for 3D imaging of vessels of the spinal cord and brain and was adapted for the analyses of murine vascular networks in our previous studies [20–24]. After contrast infusion, the vascular system is filled with a contrast agent, which can stabilize the shape of blood vessels during the preparation process and a scanning procedure involving the whole specimen. In this manner, a very detailed and fixed vascular morphology of the spinal cord of mice can be obtained. Golgi staining is a classical method allowing the detailed morphological features of neurons to be visualized based on the deposition of metal precipitates in a random set of neurons. However, the Golgi method is restricted by tissue transparency, meaning that imaging is typically limited to 200 μm in depth, which largely precludes their use in 3D visualization of large specimens [25–27]. In our study, we combined Golgi staining and angiography to visualize the 3D morphology of the spinal neurovascular system using SRμCT. We developed a new strategy that enables reliable and automated rendering of 3D integrated neurons and capillaries in the mouse thoracic spinal cord in the same image. Moreover, the quantification of blood vessels, neurons (including the soma, dendrites, and axons), and nerve fibers were also obtained in our study. Our 3D imaging method provides information leading to a comprehensive understanding of the neurovascular microstructure of the CNS and allowing thorough investigation of the effects of certain treatments in models of neurological diseases.

## Materials and Methods

### Experimental Animals and Ethics Statement

All animal protocols were approved by the Animal Ethics Committee of Central South University (approval No. 20180218). Animal care and use were conducted under the guidelines of the Administration Committee of Affairs Concerning Experimental Animals in Hunan Province, China. A total of 24 adult male C57/BL6 mice (20–23 g body weight) were obtained from the Animal Center of Central South University and kept in a temperature-controlled room with a 12 h light/dark cycle and with free access to food and water. All the mice were randomly divided into four groups of 6 each: a 3D vascular imaging group, a 3D neural imaging group, and a 3D neurovascular imaging group for the SRμCT study, and an immunofluorescence staining group.

### Perfusion and Tissue Processing

Mice were euthanized after deep anesthesia with ketamine (100 mg/kg, i.p.)/xylazine (10 mg/kg, i.p.) and transcardially perfused with 0.9% NaCl and 4% paraformaldehyde for fixation. Then, the T10–L2 segments were removed and postfixed in 4% paraformaldehyde for 24 h. Next, the segments were dehydrated in 30% sucrose/phosphate-buffered saline (PBS) overnight. After dehydration, the segments were divided into two equal parts. One part was sectioned in the sagittal plane and the other was cut coronally (at 30 μm) on a freezing microtome (HistoCore BIOCUT, Shanghai, China). The sections were stored at −20°C in cryoprotectant [ethylene glycol, glycerol, 0.1 mol/L phosphate buffer (pH 7.4), 1:1:2 by volume] until use for immunohistochemistry.

### Immunohistochemistry and Microscopy

Free-floating spinal cord sections were washed three times in PBS, followed by 1 h of blocking in PBS with 5% donkey serum. Then, the sections were incubated in primary antibodies for 24 h at 4°C: mouse anti-NeuN antibody, clone A60, Alexa Fluor®488 (1:500; Millipore Sigma, MAB377B) for neuron visualization; rabbit anti-neurofilament heavy polypeptide (1:500; Abcam, ab8135) for neurofilament visualization; and rabbit anti-CD31 (1:200; R&D Systems) for vessel visualization. After primary antibody incubation, the sections were washed three times in PBS and incubated in PBS with 5% donkey serum for 1 h. Next, these sections were incubated with the following secondary antibodies for 1 h at room temperature (RT): donkey anti-rabbit Alexa Fluor 488 (neurofilament) and donkey anti-rabbit Cy3 (vessel). Cell nuclei were stained with DAPI (4’6’-diamidino-2-phenylindole) (1 mg/mL, Sigma-Aldrich). After the above procedures, these stained sections were washed three times in PBS. Finally, the slides were mounted with Vectashield mounting medium (Vector Labs).

Images were acquired with a Zeiss Axioplan 2 upright epifluorescence microscope, Leica TCS SP8X, or a Zeiss LSM510 META confocal microscope (Leica SP8 X Confocal Microscope, Boston, USA). Image processing and assembly were performed with ImageJ/Fiji (version 2.0.-RC-43/1.51 g). To quantify the distribution of the 3D neurovascular network of gray matter in histological sections, 225 µm × 150 µm × 15 µm region located in the dorsal horn (DH), ventral horn (VH), and intermediate gray matter (IGM) were selected for measurements and comparisons.

## Preparation of Spinal Cord Samples for SRμCT Measurements

### Neural Imaging Group

For neural network visualization, to remove the blood in the vessels and achieve good staining results, mice were euthanized and perfused transcardially with artificial cerebrospinal fluid [ACSF (in mmol/L): NaCl 125, KCl 3, CaCl_2_ 2.5, MgSO_4_ 1.3, NaH_2_PO_4_ 1.25, NaHCO_3_ 26, glucose 13, in 1000 mL double-distilled-H_2_O (dd-H_2_O)] until all the blood was flushed out, which took up to 5 min. And then the spinal cord was removed with a blade. The isolated spinal cord tissue was processed by impregnation with Golgi-Cox solutions (FD Rapid GolgiStain Kit, FD NeuroTechnologies, Inc., Catalog: PK401). All glass and plastic bottles were rinsed with fresh dd-H_2_O before use. First, the tissue was immersed in the impregnation solution (solution A/B, 1:1, FD NeuroTechnologies) and stored at RT for two weeks in the dark. The impregnation solution was replaced after the first 12 h of immersion. Then, the tissue was rinsed in dd-H_2_O for 24 h. The dd-H_2_O was replaced after the first 12 h of immersion. Second, the tissue was transferred into solution C (FD NeuroTechnologies) and stored at RT in the dark for 48 h. Solution C was replaced at least once after the first 24 h. Finally, the tissue was rinsed in dd-H_2_O for 24 h. After Golgi-Cox impregnation, the stained spinal cord was dehydrated in sequential ethanols (50%, 70%, 80%, 90%, 95%, and 100%) at RT and maintained in methyl salicylate until SRμCT assessment.

### Vascular Imaging Group

To visualize the 3D vasculature, mice were euthanized and perfused transcardially with heparinized saline followed by Microfil®, a low-viscosity radio-opaque polymer (Flow Tech, Inc., Carver, USA), as previously described [28]. One centimeter of the thoracolumbar cord at T10–L2 was harvested, fixed in 4% paraformaldehyde for 24 h, dehydrated in sequential ethanols (50%, 70%, 80%, 90%, 95%, and 100%) at RT, and maintained in methyl salicylate (Millipore Sigma, M6752-1L) until use.

### Neurovascular Imaging Group

For simultaneous neurovascular network visualization, mice were euthanized and perfused transcardially with ACSF followed by filtered Microfil, and then the T10–L2 spinal segments were quickly removed. The isolated segments were processed by the above Golgi-Cox impregnation protocol. The dehydration process was the same as the that for the neural imaging group. After SRμCT assessment, the specimen was further cut into thick sections (100 μm) and observed under a stereomicroscope.

## High-Resolution SRμCT Detection and Projection Image Collection

Specimens were imaged at the BL13W1 beamline of the Shanghai Synchrotron Radiation Facility (SSRF, China). Spinal cord samples were placed and fixed in the middle of the sample stage and imaged using SRμCT. The distance between the detector and the sample was adjusted to 3 cm. The samples for vasculature visualization and neural network visualization were scanned with photon energies of 16.0 keV and 20.0 keV, respectively. The size of the beam was approximately 45 mm (horizontal) × 5 mm (vertical), and a double-crystal monochromator with Si (111) and Si (311) crystals was used to monochromatize the X-rays. After penetration through the sample, the X-rays were converted into visible light by a cleaved Lu_2_SiO_5_:Ce single-crystal scintillator (10 μm thickness). Projections were magnified by diffraction-limited microscope optics (4×/10× magnification for neural/neurovascular network visualization and 4× magnification for vasculature visualization). Then, it was digitized with a high-resolution detector (ORCA Flash 4.0 Scientific CMOS, Hamamatsu K.K., Shizuoka Prefecture, Japan) with an optical equivalent pixel size physical pixel size of 1.625 μm × 1.625 μm (4× magnification)/0.65 μm × 0.65 μm (10× magnification). The samples were rotated continuously during the scan, and 900 projection images were captured over 180° of rotation. Eventually, the 900 projection images were processed by PITRE software and transformed into 2D attenuation-contrast imaging (ACI) images. The exposure time for each projection image was set to 600 ms for vasculature and 1 s for neural/neurovascular network visualization. During the SRμCT scanning, 20 light-field images and five dark-field images were also collected during each acquisition procedure to correct for the electronic noise and variations in the X-ray source brightness.

## 3D Image Reconstruction and Quantitative Determination

All projected tomographic images were transformed into 2D ACI slice sections using the software (Phase-sensitive X-ray Image processing and Tomography Reconstruction, PITRE) developed by the SSRF to perform a direct filtered back-projection algorithm [29]. Then, all the 2D slices of the spinal cord were processed by Amira software (version 6.01, FEI, USA) to obtain the reconstructed 3D images [30]. Depending on the magnitude of X-ray absorption by the neurons and vasculature, differences in the gray values among neurons, vasculature, and their surrounding tissue were determined and segmented, and microstructures of interest with a length of 3 mm for vascular analysis and a length of 1 mm for neural network analysis were extracted from the 3D models by segmentation. To quantify morphological differences between neurons in the DH (laminae III–VI) and VH (laminae VIII–IX), five representative neurons were selected from each and the soma volume and neurite length were measured. To analyze the spatial distribution of the 3D neurovascular network of gray matter in the thoracolumbar spinal cord, the neuron and vasculature in a 225 µm × 150 µm × 225 µm volume from the VH, DH, and IGM (lamina VII) were selected from each mouse for quantitative evaluation. The 3D-rendered data were analyzed with Image-Pro Analyzer 3D (version 7.0, Media Cybernetics, Inc., Bethesda, USA) to obtain quantitative data for the neural and vascular structures [31].

## Statistical Analysis

All quantitative data are presented as the mean ± SD. The 3D morphologic parameters of the vascular and neural network data conformed to a normal distribution. Student's *t*-test was used for soma volume comparison. One-way ANOVA was used for vessel volume/neuron volume comparison. All analyses were carried out using SPSS version 19.0 (IBM Corp., Armonk, USA), and *P*-values <0.05 were considered to indicate statistical significance.

## Results

### Visualization of Nerve Fibers in White Matter and Neurons in Gray Matter Using High-Resolution SRμCT *Versus* Histological Methods

**Fig. 1:**
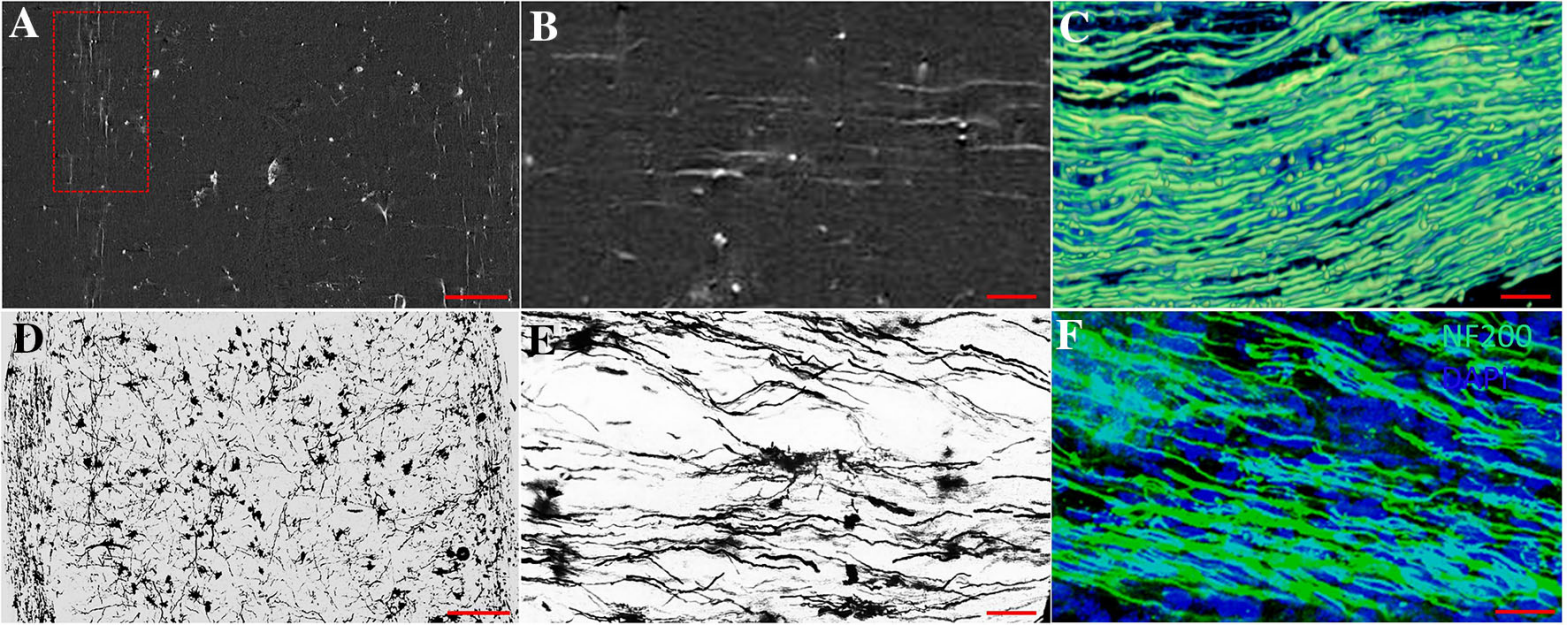
2D and 3D visualization of nerve fibers in the spinal cord. **A** Attenuation-contrast imaging (ACI) slice of the Golgi-stained spinal cord in a normal mouse (scale bar, 100 µm). **B** Local magnification of the region of interest denoted by the red frame in **A** (scale bar, 15 µm). **C** 3D tomography of white matter fiber tracts (scale bar, 15 µm). **D** Image of 100 µm thick section stained by the Golgi method (scale bar, 100 µm). **E** White matter fiber tracts labeled by the Golgi method (scale bar, 15 µm). **F** Image of white matter fiber tracts labeled with NF200 immunofluorescence (scale bar, 15 µm). Green, NF200; blue, DAPI.

**Fig. 2:**
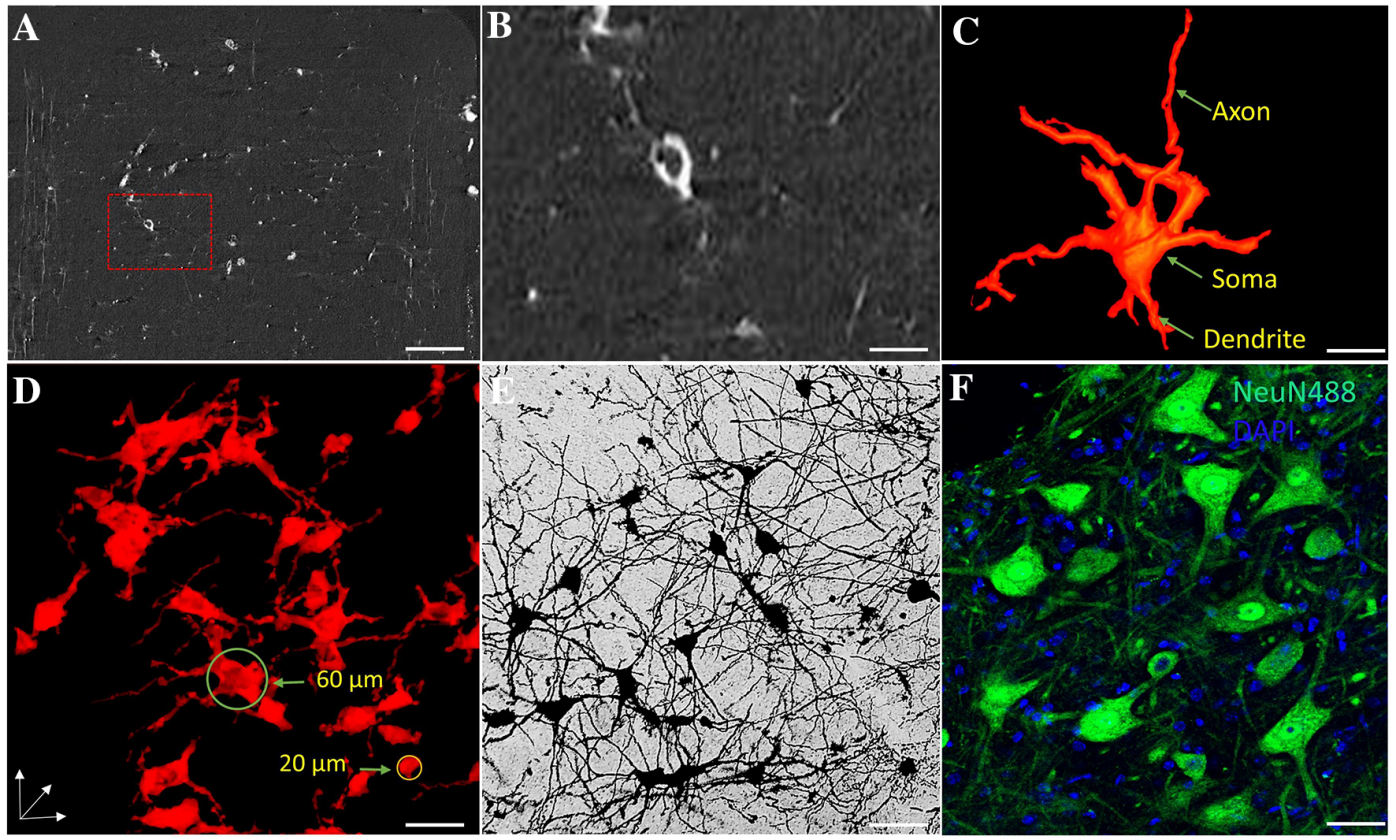
2D and 3D imaging of neurons in the spinal cord. **A** ACI slice of the Golgi-stained spinal cord in a normal mouse (scale bar, 120 µm). **B** Local magnification of the region of interest denoted by the red frame in **A** (scale bar, 50 µm). **C** 3D tomography of a single neuron (scale bar, 75 µm). **D** 3D tomography of the neuronal network (scale bar, 60 µm). **E** Golgi-stained neuronal network (scale bar, 80 µm). **F** NueN488 immunofluorescence image of neurons (scale bar, 70 µm). Green, NeuN488; blue, DAPI.

We visualized a nerve fiber using immunohistochemistry and a combination of SRμCT and Golgi staining. First, a positive signal of nerve fiber in 2D ACI slices was defined as an area with high gray values in white matter (Fig. 1A, B). Then, after 3D reconstruction, 3D nerve fibers generated by SRμCT were well depicted in the white matter area (Fig. 1C). To verify the reliability of this technique, we compared the 3D nerve fiber image with 2D images of nerve fibers visualized by Golgi and immunofluorescence staining (Fig. 1D–F). Our results showed that the distribution, arrangement, and sizes of the fibers in the 3D image were similar to those in the 2D image. Furthermore, because the neuronal architecture was also stained with heavy metals, an individual neuron could be identified as having high-absorbing structures in the gray matter. Our method distinguished neurons from unstained areas (Fig. 2A, B). After reconstruction, the 3D morphology of a single neuron was comprehensively presented, in which the soma, axon, and dendrites were well visualized. As shown in Fig. 2C, the axon tended to be long, untapered, and unbranched, whereas dendrites were shorter, tapered, and highly branched. Our measurements showed that the diameter of the neurons in the VH ranged from 20 to 60 μm (Fig. 2D). In addition, we compared the 3D images of neurons acquired by SRμCT with the 2D images of neurons visualized by Golgi and immunofluorescence staining. Although the neuronal architecture in the 3D images was morphologically consistent with that in 2D histological sections, neurons in 3D were visualized in their entirety in contrast to the results with the histological method (Fig. 2D–F). Our results showed that 3D nerve fibers and neurons were clearly visualized by our method based on combination of SRμCT and the Golgi staining technique.

### Visualization of Angioarchitecture in the Mouse Thoracolumbar Spinal Cord Using High-resolution SRμCT *Versus* Histological Methods

**Fig. 3:**
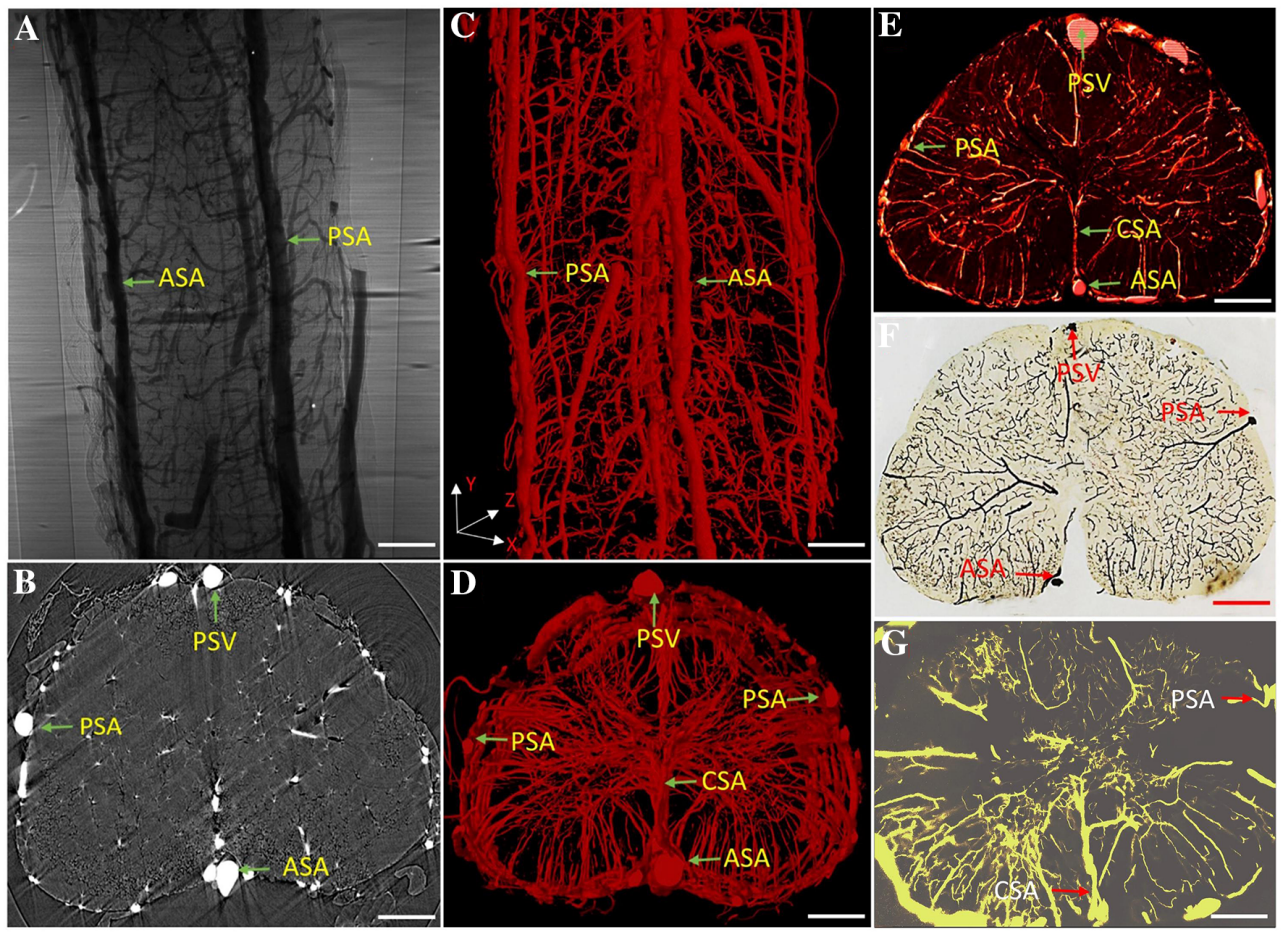
2D and 3D imaging of vasculature. **A** Synchrotron radiation in-line ACI projection of the spinal vessels in mice. **B** ACI cross-sectional slice of the thoracolumbar spinal cord. **C–D** 3D tomography of the thoracolumbar angioarchitecture. **E** Image of a virtual section 50 µm thick. **F** Image of a histological section (60 µm thick) with vessels filled by contrast agent. **G** Image of a histological section (200 µm thick) filled with Microfil agent imaged by stereomicroscope (scale bar, 250 µm). ASA, anterior spinal artery; CSA, central sulcus artery; PSA, posterolateral spinal arteries; PSV, posterior spinal vein.

**Fig. 4:**
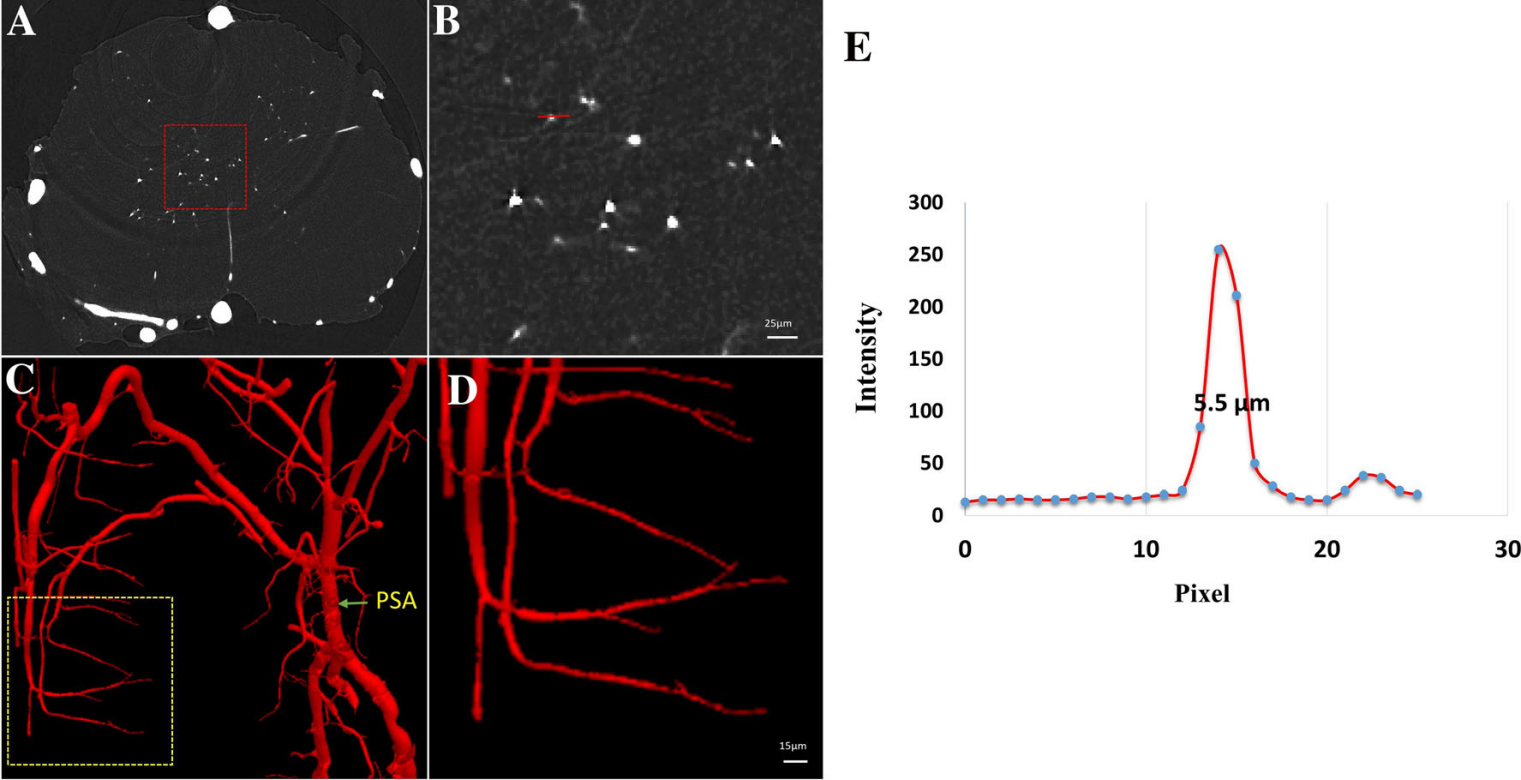
3D imaging of capillaries of the spinal cord. **A** ACI virtual cross-sectional slice. **B** Local magnification of the region of interest denoted by the red frame in **A** (scale bar, 25 µm). **C** 3D tomography of capillaries. **D** Local magnification of the region of interest denoted by the yellow frame in **B** (scale bar, 15 µm). **E** Profile along the red line in **B**. PSA, posterolateral spinal arteries.

We applied the combination of high-resolution SRμCT and angiography to create maps of the detailed 3D angioarchitecture of the mouse thoracolumbar spinal cord. Due to the high absorbance efficiency of the contrast agent (Microfil), blood vessels were clearly discernible on the ACI projection image and were distinguishable from the surrounding tissue (Fig. 3A). In ACI slices, areas with high gray values were vessels with opaque contrast on the X-ray images (Fig. 3B). Second, after 3D reconstruction, the thoracolumbar spinal cord was selected for display. As shown in Fig. 3C, D, 3D high-resolution images of the intramedullary vessels clearly presented the detailed morphology of the central radial vessels entering the gray matter from the anterior spinal artery (ASA). The location and shape of the ASA, central sulcus artery (CSA), posterolateral spinal arteries (PSAs), and posterior spinal vein (PSV) were perfectly delineated in the 3D images. More importantly, we compared the virtual and histological sections to indicate the reliability of using SRμCT in vascular assessment. We first compared the reconstructed 3D virtual sections (50 μm thick) with histological sections (60 μm thick) in which the blood vessels were filled with contrast agent (Fig. 3E, F). Then, we also compared the reconstructed coronal 3D images (Fig. 3D) with 200 μm thick histological sections imaged using a stereomicroscope (Fig. 3G). The results of the 3D-rendered images showed good consistency with the histological section images, without sacrificing the integrity of the vascular structure by sectioning during sample preparation. To visualize capillaries, we used a high-resolution detector with an optical equivalent pixel size of 1.625 μm × 1.625 μm and found that, in virtual cross-sectional slices, the boundaries of microvessels with contrast perfusion were detectable (Fig. 4A, B). The grey level of the line profiles marked in Fig. 4B is illustrated in Fig. 4E. After 3D reconstruction, our results showed that capillaries as small as 5.5 μm in diameter were detectable in 3D images (Fig. 4C, D). Without sacrificing quality anywhere in the sample, SRμCT provide detailed 3D morphological information of the complicated angioarchitecture in the spinal cord as a color-rendered stereostructure.

### Simultaneous Visualization of Neural and Vascular Structures in the Mouse Spinal Cord Using High-Resolution SRμCT *Versus* Histological Methods

**Fig. 5:**
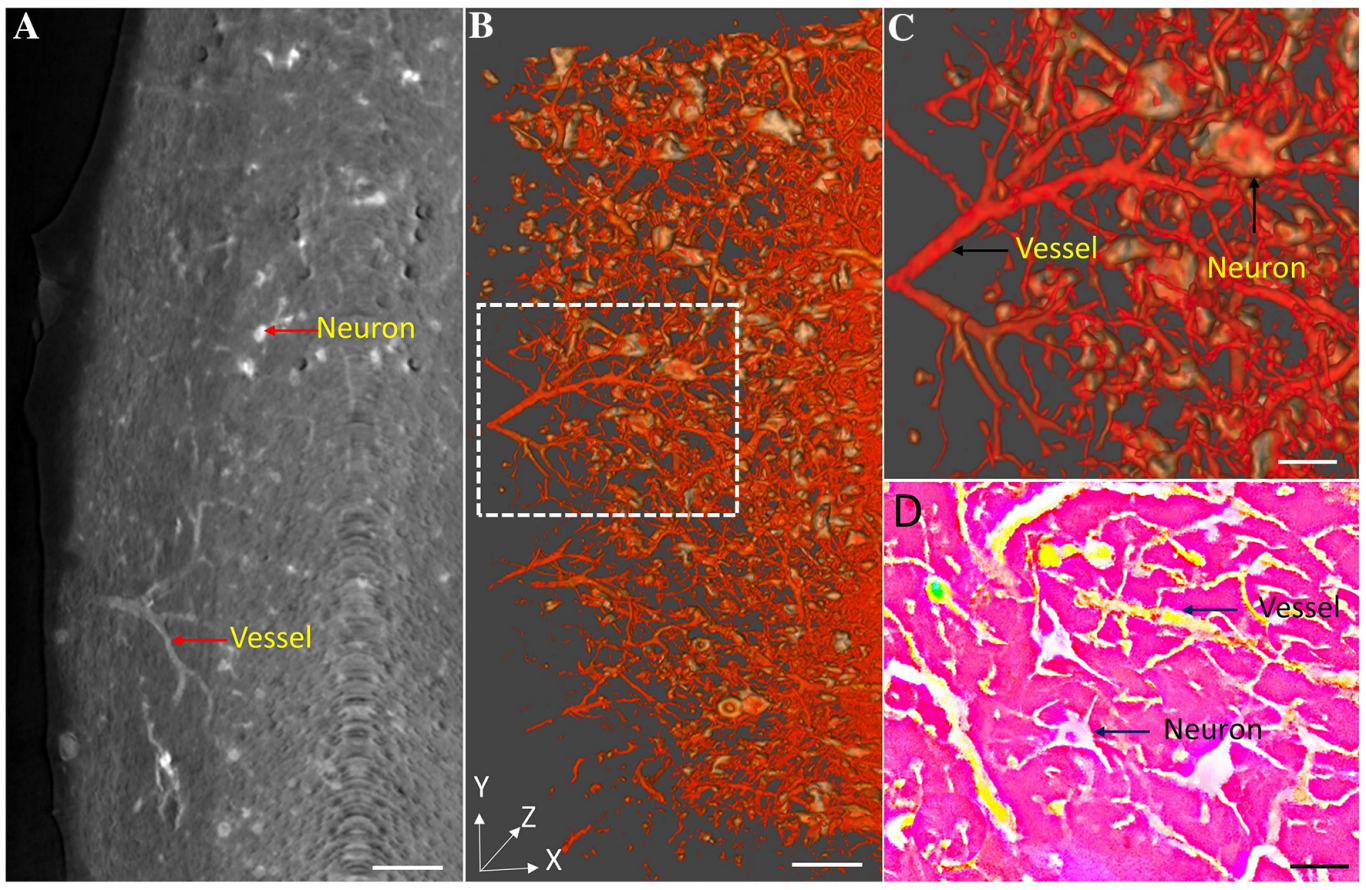
Simultaneous imaging of the neurovascular network in the spinal cord. **A** ACI slice of Golgi-stained spinal cord with vessels filled by the contrast agent Microfil (scale bar, 100 µm). **B** 3D image of the neurovascular network (scale bar, 100 µm). **C** Local magnification of the region of interest denoted by the white frame in **B** (scale bar, 50 µm). **D** Image of the intramedullary vessels and neurons obtained using the stereomicroscope (scale bar, 50 µm; yellow: vessels; white: neurons).

To acquire simultaneous 3D images of neurovascular structures, we combined Golgi staining, angiography, and SRμCT to simultaneously image neural and vascular networks in the mouse spinal cord. Positive signals of neurons and vessels were identified in 2D ACI slices (Fig. 5A). Neurons and microvessels were visualized after 3D reconstruction, and neurons were surrounded by abundant capillaries (Fig. 5B, C). To test the reliability of using SRμCT for neurovascular morphology detection, the scanned spinal cords were cut into slices, and visualized under a stereomicroscope. The results showed that the detected neurons and vessels could also be seen in the corresponding histological sections (Fig. 5D). These results show that combined Golgi staining, angiography, and SRμCT simultaneously visualize the neural and vascular structure of the spinal cord.

### Quantification of Morphological Differences Between Neurons in the DH (Laminae III–VI) and VH (Laminae VIII–IX)

**Fig. 6:**
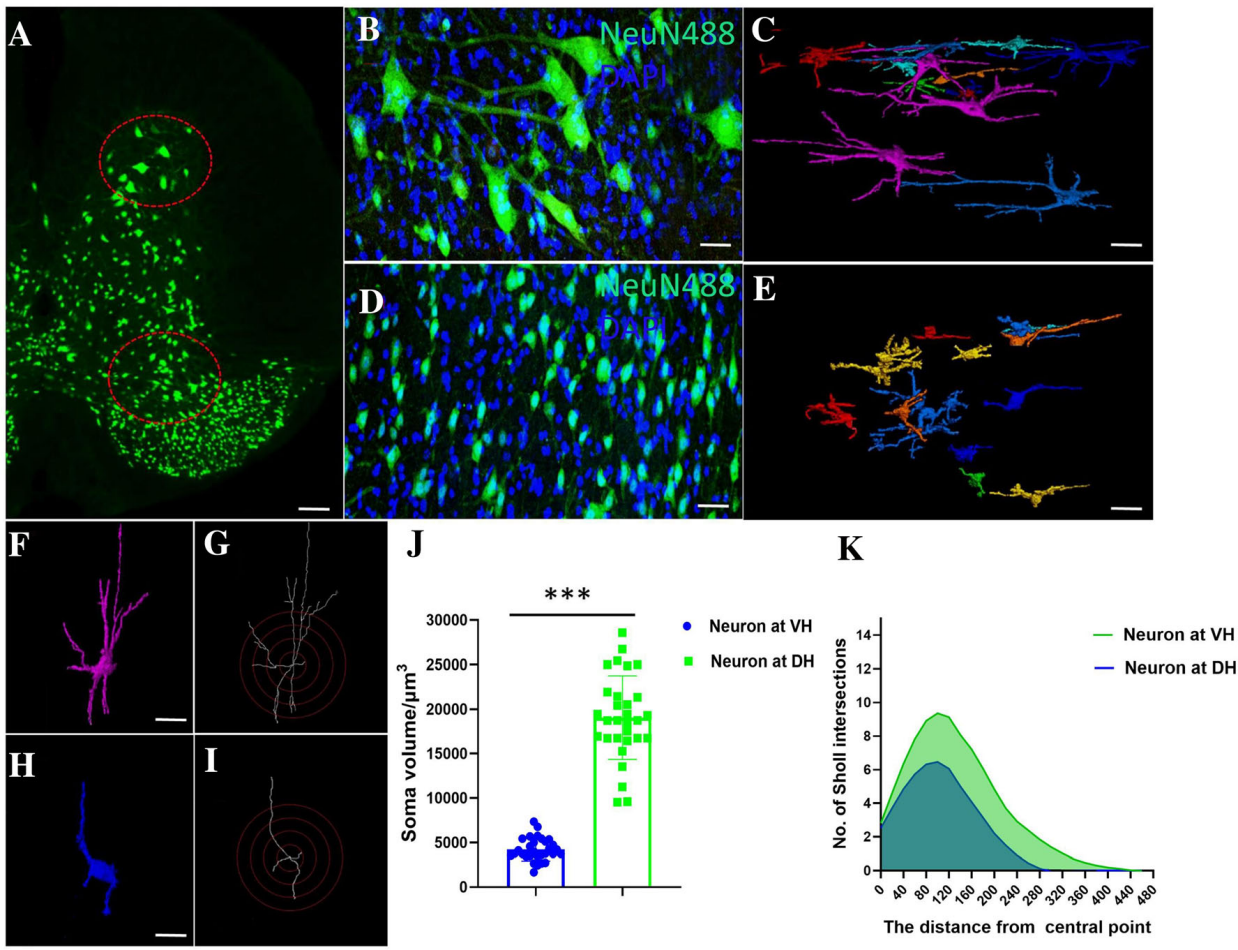
Quantification of neuronal architecture of the spinal cord. **A** Cross-sectional histological image of a 30 µm histological section stained by NeuN488 immunofluorescence (scale bar, 100 µm). **B** Histological image of the neural network in the VH (scale bar, 30 µm; green, NeuN488; blue, DAPI). **C** 3D tomography of the neural network in the VH (scale bar, 50 µm). **D** Histological image of neurons in the DH (scale bar, 30 µm; green, NeuN488; blue, DAPI). **E** 3D tomography of the neural network in the DH (scale bar, 50 µm). **F** Representative 3D neuron in the VH (scale bar, 60 µm). **G** Example image for quantification of Sholl intersections of neurons in the VH. **H** Representative 3D neuron in the DH (scale bar, 60 µm). **I** Example image for quantification of Sholl intersections of neurons in the DH. **J** Statistics of 3D soma volume in the VH and DH (*n* = 5 × 6). **K** Quantitative statistical results of the number of Sholl intersections (*n* = 5 × 6, ***P <0.001).

We conducted a systematic quantitative evaluation of neuronal morphology in the mouse thoracolumbar spinal gray matter. First, we compared the 3D images of neurons generated by SRμCT in the VH and DH with the corresponding 2D images of neurons visualized by immunofluorescence. The morphological information obtained by the two methods was consistent (Fig. 6A–E). Then, quantitative comparative analysis was performed on neurons in the VH and DH. The results showed that the neuronal soma volume in the VH was larger than that in the DH (*P* <0.01). The results of Sholl analysis showed that the length and coverage of the neurites of VH neurons were also greater than the corresponding parameters of neurons in the DH (*P* <0.01) (Fig. 6F–K). So, neurons in the VH are significantly larger than neurons in the DH. The combination of SRμCT and Golgi staining has an advantage in the quantification of detailed 3D neuronal architecture

### Quantitative Characterization of the Distribution of the 3D Neurovascular Network in the Gray Matter of the Thoracolumbar Spinal Cord

**Fig. 7:**
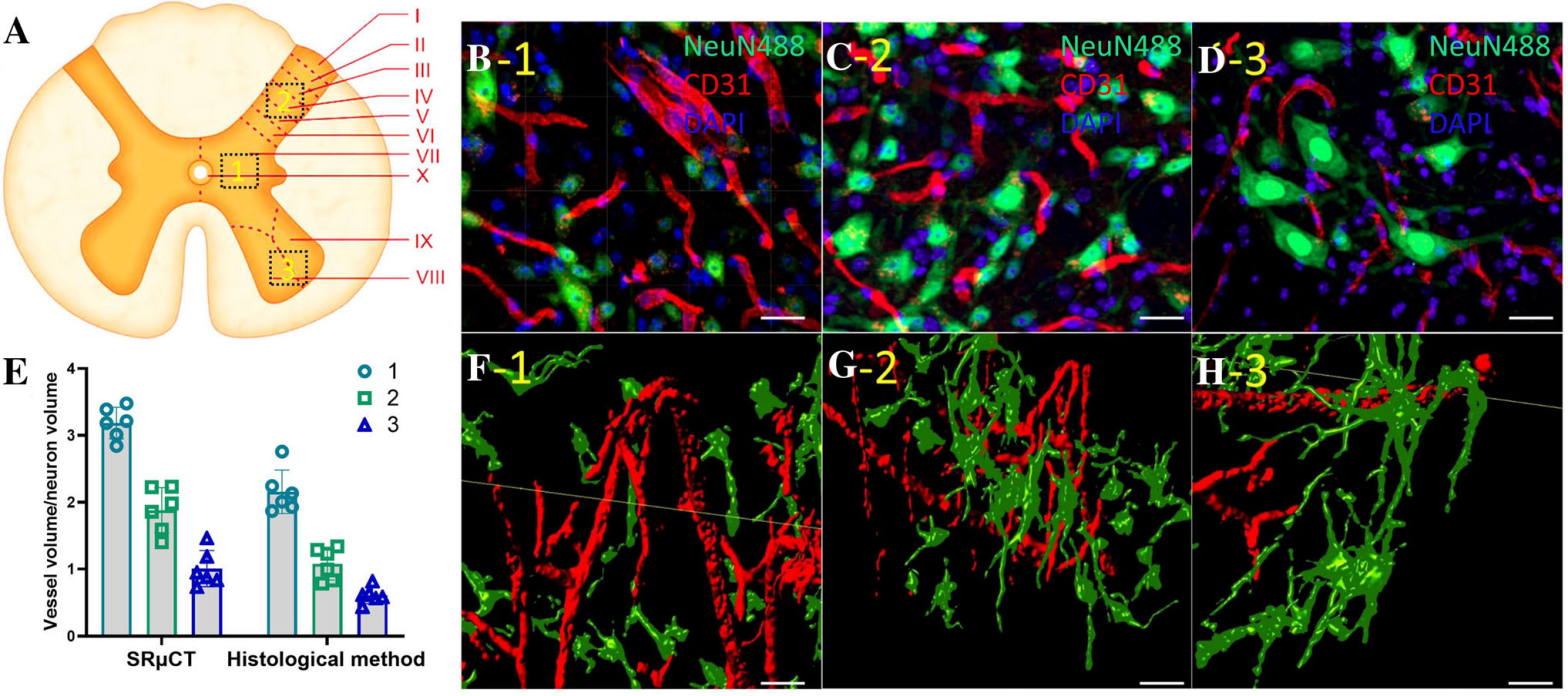
Quantification of neurovascular network. **A** Schematic of a spinal cord cross-section. **B–D** Histological images of neurons and vessels at locations 1–3 denoted by the black frames (225 µm × 150 µm × 225 µm) in **A** (scale bars, 30 µm; green, NeuN488; red, CD31; blue, DAPI). **F–H** Representative 3D images of the neurovascular network at the location of 1–3 denoted by the black frame in **A** (scale bars, 30 µm). **E** Quantitative results of 3D vessels/neurons from the locations in **A** (*n* = 6).

To better understand the distribution of neurons and vessels in the 3D space at T10 in the spinal cord, we quantified and compared the ratios of blood vessel volume/neuron volume (BV/NV) in three areas: the IGM (lamina VII), VH (laminae VIII–IX), and DH (laminae III–VI)) (Fig. 7A) from 6 wild-type mice. Representative 3D images of the region of interest of the neurovascular network in the three areas were selected (Fig. 7F–H). We found that the BV/NV ratios in the IGM, VH, and DH were 3.19 ± 0.21, 1.89 ± 0.34, and 1.01 ± 0.27, respectively. In addition, we found a similar neural and vascular distribution using the histological method (Fig. 7B–D). The statistical results are presented in Fig. 7E. The results show that the combination of SRμCT, Golgi staining, and angiography has an advantage in the quantification of detailed 3D neural and vascular structure.

## Discussion

Morphological visualization is of vital importance for biological and medical research, as there is a close correlation between microscopic neurovascular changes and disorders of the CNS. In this study, we demonstrated that our 3D imaging method based on SRμCT allows for comprehensive high-resolution 3D imaging of the vasculature and the neural network and even permits simultaneous 3D visualization of the neural and vascular microstructure in the mouse spinal cord. In addition, the 3D imaging results were consistent with the widely-used 2D histological method. Our results confirmed the accuracy and reliability of the SR-based ACI method combined with Golgi staining and angiography, which can be used to explore the 3D morphological characteristics and distribution of the vasculature and neural systems in wide spatial regions of the spinal cord without sectioning.

First, for 3D imaging of the neural network *via* the combination of Golgi staining and SRμCT, we achieved not only the comprehensive 3D visualization of neurons in gray matter and entire nerve fibers in white matter but also 3D quantification of neuronal architecture based on integrated neurons with somas, axons, and dendrites. The consistency of 3D images with 2D histological sections indicated the reliability of our 3D morphological results. It has been reported that the 3D imaging of neurons in an unstained mouse spinal cord can be achieved using SRXPCT [8–10]. However, the authors only visualized a part of the neuronal structure, especially the soma; morphological information on axons and dendrites is very limited. Recently, although some researchers obtained 3D images of large neurons (e.g., Purkinje cells) after optimization of the sample preparation and parameter scanning [11, 12, 32], the morphological integrity of Purkinje cells obtained by SRXPCT was not as immaculate as that captured by the Golgi method [33]. Due to the small size of axons and dendrites and the similarity of their refractive index with that of the extracellular matrix, it was difficult to segment and visualize the integrated neuronal architecture. In the present study, the Golgi staining method labeled the soma, axon, and dendrite with heavy metals, which precluded the previous issue and increased the contrast between neurons and the extracellular matrix. Hence, an integrated neuron with a soma, axon, and dendrites could be depicted in our 3D images. Furthermore, our quantitative results were more consistent than the results obtained in the previous study [9]. In addition to accurately measuring the size of the motor neurons in the VH as in Bukreeva’s study [9], we further identified the difference in neuronal architecture between neurons in the VH and neurons in the DH *via* our method. In our 3D images, the volume of the soma and the neurite coverage of neurons in the DH were significantly smaller than those of VH neurons. According to our results, Golgi staining optimized the visualization of the neural structure based on SRμCT. The combination of Golgi staining and SRμCT had the advantages in the measurement of detailed individual neuronal architecture.

Second, the 3D vasculature of the thoracolumbar segment of the mouse spinal cord and the “butterfly” shape of intramedullary vessels were vividly visualized by our method, which was difficult to accomplish using the classical histological method. Capillaries as small as 5–10 μm in diameter were identified in our results. In addition, by comparing the virtual section with a commonly used histological section, we found that the location of large vessels and the distribution of microvessels were consistent. Furthermore, the 3D image could offer morphological details of the vasculature superior to 2D histological sections. Massimi *et al*. and Fratini *et al*. also reported that they achieved 3D imaging of the mouse intramedullary vascular network using SRXPCT [8, 34]. Unfortunately, they only visualized large vessels, such as the ASA and CSA, and very few microvessels in the spinal cord. The microvessels, especially capillaries without the support of vascular smooth muscle, may easily become deformed, shrink, and even collapse during sample preparation and scanning, which leads to a possibly inaccurate interpretation in subsequent quantitative analysis. In our study, we explored several techniques to solve this problem and eventually achieved reliable and stable imaging of detailed angioarchitecture *via* the combination of SRXPCT and angiography.

Third, for 3D neurovascular morphology visualization, we successfully achieved simultaneous 3D imaging of the neural and vascular structures and optimized 3D visualization. Although the simultaneous 3D visualization of neurons and vessels in unstained CNS tissues has been achieved by high-resolution SRXPCT, the detailed morphological information of axons and dendrites was absent in most of these studies [8, 10, 12, 35]. In this research, we present the neuronal and microvascular network in the same image with high resolution and fine quality, which allows the neurovascular network to be measured in a 3D reconstruction. We quantified the distribution of the neurons and vessels in the same 3D space of the spinal cord and found that their distribution was similar to the results in 2D histological sections. Moreover, we discovered that the BV/NV ratio in the IGM was larger than that in the VH and DH neurons, which was not previously been reported. Based on the above findings, combining Golgi staining and angiography simultaneously visualize the neuronal and vascular networks in the mouse spinal cord based on the SRμCT imaging technique. As our established method could reveal structural changes in various CNS disease models, especially diseases with an entangled relationship between vascular and neuronal systems.

However, there are still some limitations in our method. First, the process of Golgi impregnation requires two weeks, which is relatively time-consuming. Second, the limitation of Golgi staining and angiography restricts our access to more subtle morphologies, such as the subcellular structures of neurons and vascular wall cells.

## Conclusion

The combination of SRμCT, Golgi staining, and angiography can simultaneously reveal the 3D neurovascular morphology of the spinal cord without a destructive slicing process and serves as a reliable method to simultaneously quantify the vasculature and neural networks.

## Supplementary Information

Below is the link to the electronic supplementary material.

Supplementary file1 (PDF 234 kb)

## References

[1] McKinley W, Santos K, Meade M, Brooke K (2007). Incidence and outcomes of spinal cord injury clinical syndromes. J Spinal Cord Med.

[2] David G, Mohammadi S, Martin AR, Cohen-Adad J, Weiskopf N, Thompson A (2019). Traumatic and nontraumatic spinal cord injury: pathological insights from neuroimaging. Nat Rev Neurol.

[3] Saliani A, Zaimi A, Nami H, Duval T, Stikov N, Cohen-Adad J (2019). Construction of a rat spinal cord atlas of axon morphometry. Neuroimage.

[4] Rauschenbach L (2020). Spinal cord tumor microenvironment. Adv Exp Med Biol.

[5] Helmchen F, Denk W (2005). Deep tissue two-photon microscopy. Nat Methods.

[6] Wu J, He Y, Yang Z, Guo C, Luo Q, Zhou W (2014). 3D BrainCV: simultaneous visualization and analysis of cells and capillaries in a whole mouse brain with one-micron voxel resolution. Neuroimage.

[7] Massimi L, Bukreeva I, Santamaria G, Fratini M, Corbelli A, Brun F (2019). Exploring Alzheimer's disease mouse brain through X-ray phase contrast tomography: From the cell to the organ. Neuroimage.

[8] Fratini M, Bukreeva I, Campi G, Brun F, Tromba G, Modregger P (2015). Simultaneous submicrometric 3D imaging of the micro-vascular network and the neuronal system in a mouse spinal cord. Sci Rep.

[9] Bukreeva I, Campi G, Fratini M, Spano R, Bucci D, Battaglia G (2017). Quantitative 3D investigation of neuronal network in mouse spinal cord model. Sci Rep.

[10] Cedola A, Bravin A, Bukreeva I, Fratini M, Pacureanu A, Mittone A (2017). X-ray phase contrast tomography reveals early vascular alterations and neuronal loss in a multiple sclerosis model. Sci Rep.

[11] Topperwien M, van der Meer F, Stadelmann C, Salditt T (2018). Three-dimensional virtual histology of human cerebellum by X-ray phase-contrast tomography. Proc Natl Acad Sci U S A.

[12] Topperwien M, Markus A, Alves F, Salditt T (2019). Contrast enhancement for visualizing neuronal cytoarchitecture by propagation-based X-ray phase-contrast tomography. Neuroimage.

[13] Strotton MC, Bodey AJ, Wanelik K, Hobbs C, Rau C, Bradbury EJ (2021). The spatiotemporal spread of cervical spinal cord contusion injury pathology revealed by 3D in-line phase contrast synchrotron X-ray microtomography. Exp Neurol.

[14] Gu P, Xu ZH, Cao YZ, Liao SH, Deng QF, Yin XZ (2020). Synchrotron radiation-based three-dimensional visualization of angioarchitectural remodeling in hippocampus of epileptic rats. Neurosci Bull.

[15] Song M (2020). Imaging three-dimensional microvascular networks of brain with synchrotron radiation microangiography. Neurosci Bull.

[16] Barbone GE, Bravin A, Mittone A, Grosu S, Ricke J, Cavaletti G (2021). High-spatial-resolution three-dimensional imaging of human spinal cord and column anatomy with postmortem X-ray phase-contrast micro-CT. Radiology.

[17] Hetterich H, Willner M, Fill S, Herzen J, Bamberg F, Hipp A (2014). Phase-contrast CT: qualitative and quantitative evaluation of atherosclerotic carotid artery plaque. Radiology.

[18] Hu J, Li P, Yin X, Wu T, Cao Y, Yang Z (2017). Nondestructive imaging of the internal microstructure of vessels and nerve fibers in rat spinal cord using phase-contrast synchrotron radiation microtomography. J Synchrotron Radiat.

[19] Barbone GE, Bravin A, Mittone A, Kraiger MJ, Hrabe de Angelis M, Bossi M (2020). Establishing sample-preparation protocols for X-ray phase-contrast CT of rodent spinal cords: Aldehyde fixations and osmium impregnation. J Neurosci Methods.

[20] Hu J, Cao Y, Wu T, Li D, Lu H (2014). High-resolution three-dimensional visualization of the rat spinal cord microvasculature by synchrotron radiation micro-CT. Med Phys.

[21] Hu J, Ni S, Cao Y, Wang X, Liao S, Lu H. Comparison of synchrotron radiation-based propagation phase contrast imaging and conventional micro-computed tomography for assessing intervertebral discs and endplates in a murine model. Spine (Phila Pa 1976) 2017, 42: E883–E889. 10.1097/BRS.0000000000002110 28187077

[22] Ni S, Cao Y, Jiang L, Luo Z, Lu H, Hu J*, et al.* Synchrotron radiation imaging reveals the role of estrogen in promoting angiogenesis after acute spinal cord injury in rats. Spine (Phila Pa 1976) 2018, 43: 1241–1249. 10.1097/BRS.0000000000002629 29529001

[23] Ni SF, Luo ZX, Jiang LY, Guo Z, Li P, Xu X (2019). UTX/KDM6A deletion promotes recovery of spinal cord injury by epigenetically regulating vascular regeneration. Mol Ther.

[24] Li P, Xu Y, Cao Y, Wu TD (2020). 3D digital anatomic angioarchitecture of the rat spinal cord: A synchrotron radiation micro-CT study. Front Neuroanat.

[25] Graeden E, Sive H (2009). Live imaging of the zebrafish embryonic brain by confocal microscopy. J Vis Exp.

[26] Castano P, Gioia M, Barajon I, Rumio C, Miani A (1995). A comparision between rapid Golgi and Golgi-Cox impregnation methods for 3-D reconstruction of neurons at the confocal scanning laser microscope. Ital J Anat Embryol.

[27] Brakenhoff GJ, van der Voort HT, van Spronsen EA, Nanninga N (1988). 3-Dimensional imaging of biological structures by high resolution confocal scanning laser microscopy. Scanning Microsc.

[28] Cao Y, Wu T, Yuan Z, Li D, Ni S, Hu J (2015). Three-dimensional imaging of microvasculature in the rat spinal cord following injury. Sci Rep.

[29] Chen RC, Dreossi D, Mancini L, Menk R, Rigon L, Xiao TQ (2012). PITRE: software for phase-sensitive X-ray image processing and tomography reconstruction. J Synchrotron Radiat.

[30] Ian E, Zhao XC, Lande A, Berg BG (2016). Individual neurons confined to distinct antennal-lobe tracts in the heliothine moth: morphological characteristics and global projection patterns. Front Neuroanat.

[31] Yang P, Gandahi JA, Zhang Q, Zhang LL, Bian XG, Wu L (2013). Quantitative changes of nitrergic neurons during postnatal development of chicken myenteric plexus. J Zhejiang Univ Sci B.

[32] Hieber SE, Bikis C, Khimchenko A, Schweighauser G, Hench J, Chicherova N (2016). Tomographic brain imaging with nucleolar detail and automatic cell counting. Sci Rep.

[33] Kim J, Kwon N, Chang S, Kim KT, Lee D, Kim S (2011). Altered branching patterns of Purkinje cells in mouse model for cortical development disorder. Sci Rep.

[34] Massimi L, Fratini M, Bukreeva I, Brun F, Mittone A, Campi G (2016). Characterization of mouse spinal cord vascular network by means of synchrotron radiation X-ray phase contrast tomography. Phys Med.

[35] Topperwien M, van der Meer F, Stadelmann C, Salditt T (2020). Correlative X-ray phase-contrast tomography and histology of human brain tissue affected by Alzheimer's disease. Neuroimage.

