# Supplementary material for: Simultaneous 3D Visualization of the Microvascular and Neural Network in Mouse Spinal Cord Using Synchrotron Radiation Micro-Computed Tomography
Source: Neurosci Bull. 2021 Jun 19;37(10):1469–80. doi: 10.1007/s12264-021-00715-7 (PMC8490558; doi:10.1007/s12264-021-00715-7)
Supplement: Supplementary file 1 — Supplementary file1 (PDF 234 kb) [file 12264_2021_715_MOESM1_ESM.pdf]

Supplementary Materials

Supplementary Figure

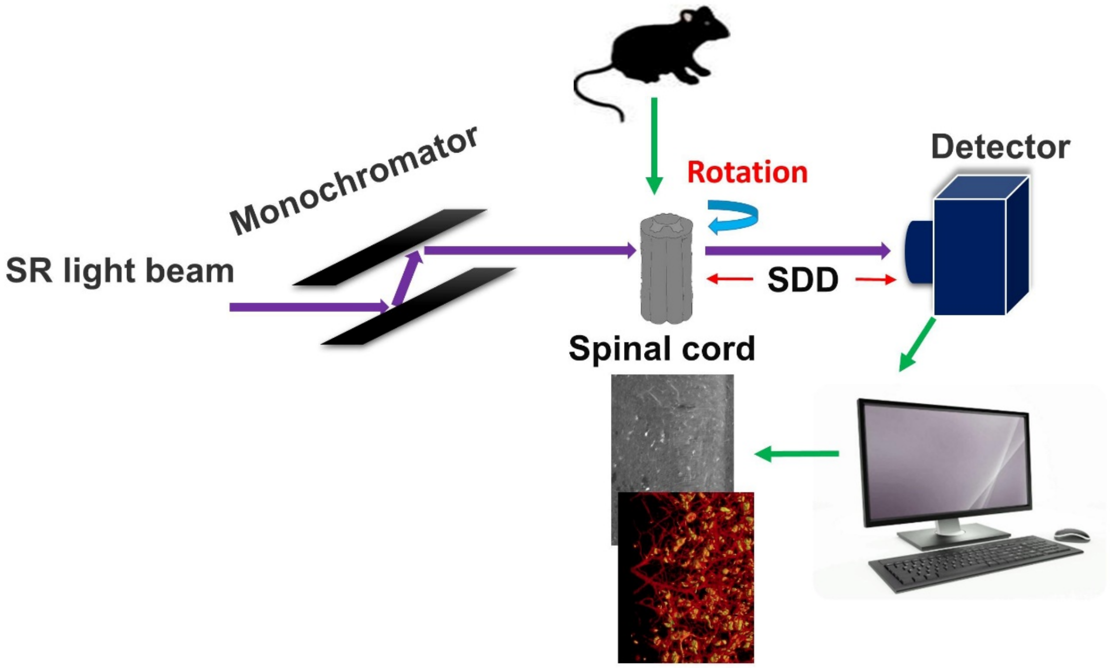

Fig. S1 Workflow of SRμCT imaging. SRμCT, synchrotron radiation micro-computed tomography; SR: synchrotron radiation; SDD: sample detector distance.
